# “Arming half-baked people with weapons!” Information enclaving among professionals and the need for a care-centred model for antibiotic use information in Uganda, Tanzania and Malawi

**DOI:** 10.1080/16549716.2024.2322839

**Published:** 2024-03-05

**Authors:** Susan Nayiga, Eleanor E MacPherson, John Mankhomwa, Fortunata Nasuwa, Raymond Pongolani, Rita Kabuleta, Mike Kesby, Russell Dacombe, Shona Hilton, Delia Grace, Nicholas Feasey, Clare I.R. Chandler

**Affiliations:** a Infectious Diseases Research Collaboration, Kampala, Uganda; b Research and Innovation Services, University of Glasgow, Glasgow, UK; c Malawi-Liverpool-Wellcome Research Programme, Blantyre, Malawi; d Kilimanjaro Clinical Research Institute (KCRI), Moshi, Tanzania; e School of Geography & Sustainable Development, University of St Andrews, St Andrews, UK; f MRC/CSO Social and Public Health Sciences Unit, University of Glasgow, Glasgow, UK; g Natural Resources Institute, University of Greenwich, Chatham, UK; h International Livestock Research Institute, Nairobi, Kenya; i The School of Medicine, University of St Andrews, St Andrews, UK; j Department of Global Health and Development, London School of Hygiene & Tropical Medicine, London, UK

**Keywords:** Antimicrobial resistance, patients, farmers, medical professionals, veterinary professionals, policy makers, drug regulators, medical knowledge, enclaving of knowledge

## Abstract

**Background:**

The overuse of antimicrobial medicines is a global health concern, including as a major driver of antimicrobial resistance. In many low- and middle-income countries, a substantial proportion of antibiotics are purchased over-the-counter without a prescription. But while antibiotics are widely available, information on when and how to use them is not.

**Objective:**

We aimed to understand the acceptability among experts and professionals of sharing information on antibiotic use with end users – patients, carers and farmers – in Uganda, Tanzania and Malawi.

**Methods:**

Building on extended periods of fieldwork amongst end-users and antibiotic providers in the three countries, we conducted two workshops in each, with a total of 44 medical and veterinary professionals, policy makers and drug regulators, in December 2021. We carried out extensive documentary and literature reviews to characterise antibiotic information systems in each setting.

**Results:**

Participants reported that the general public had been provided information on medicine use in all three countries by national drug authorities, health care providers and in package inserts. Participants expressed concern over the danger of sharing detailed information on antibiotic use, particularly that end-users are not equipped to determine appropriate use of medicines. Sharing of general instructions to encourage professionally-prescribed practices was preferred.

**Conclusions:**

Without good access to prescribers, the tension between enclaving and sharing of knowledge presents an equity issue. Transitioning to a client care-centred model that begins with the needs of the patient, carer or farmer will require sharing unbiased antibiotic information at the point of care.

## Background

Increasing use of antimicrobials for humans and animals globally is of great concern because of the growing impact of antimicrobial resistance (AMR) [1–3]. Low- and middle-income countries (LMICs) have been reported to contribute significantly to the increase in antibiotic consumption globally [4,5]. The WHO’s Global Action Plan highlights raising awareness, providing information and improving understanding of AMR as necessary first steps towards making long-term gains against AMR as a global threat [6]. Currently, messaging about AMR focuses on raising awareness of the prevalence and danger of AMR, which can be understood by prescribers and dispensers as a reason to escalate to the use of what they consider to be stronger antibiotics [7,8].

Whilst policies and regulations may be in place to restrict antimicrobial access in many LMICs, antibiotics – one particular group of antimicrobials of current concern for resistance – are often accessed readily over the counter without prescriptions [9–11]. At the same time, information about the use of antibiotics among end users is limited [12,13]. Interventions conducted in Mali, Kenya, Tanzania and England have reported potentially positive impacts of providing medicine use information to end users, suggesting that provision of targeted information through simple strategies such as leaflets can result in the safe use of medicines [14–17]. Yet, this approach remains little used, including in LMICs where formal health care can be patchy. Data tracking the Sustainable Development Goal 3.8.1, access to universal health coverage, suggests 4.5 million people globally remained without access to essential health services in 2021 [18]. Access to veterinary services is known to be very limited in low and middle-income countries, including many African settings [19]. Our study aimed to assess the acceptability and feasibility of sharing information about best practice in how to use antibiotics for residents and farmers in Uganda, Tanzania, and Malawi where increases in antibiotic use are being reported but there is limited access to antibiotic use information.

## Methods

### The enabling optimal antimicrobial use in East Africa project

Four Global Challenges Research Fund (GCRF) funded consortia collaborated in this project: the Antimicrobials in Society (AMIS) Project in Uganda, Drivers of Resistance in Uganda and Malawi (DRUM), Holistic Approach to Unravel Antibacterial Resistance in East Africa (HATUA) in Uganda, Tanzania and Kenya and Supporting the National Action Plan for Antimicrobial Resistance (SNAP-AMR) in Tanzania. Each project had undertaken research on AMR from a One Health perspective, including antimicrobial use (AMU) practices. To draw together findings on AMR and AMU across an East African context, uncover synergies that could be useful for optimising antibiotic usage (ABU), and avoid separate projects contributing to conflicting messaging throughout the region, a research cluster consisting of the four GCRF projects was formed. An emerging concern from across the consortia was the paucity of information available to end users of antibiotics. Across study settings, our teams noted a desire from residents, farmers, and some medicine sellers for more information about antibiotics [20–27]. In particular, respondents wanted to understand what conditions antibiotics can treat, which antibiotics to use and what dose and duration should be given, as well as antibiotic side effects and contraindications.

**Figure 1.**
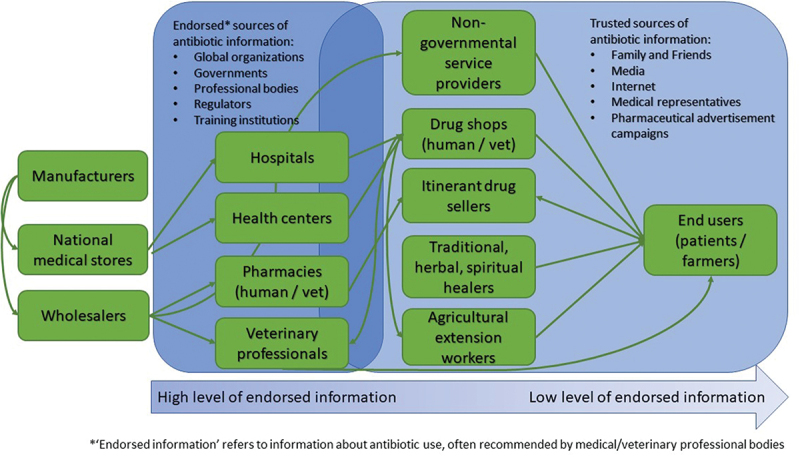
Antibiotic drug flows, information sources and desire for information.

Under the AMIS, DRUM, HATUA and SNAP-AMR projects, empirical research involving social science studies on antibiotic use were carried out involving different levels of health and food production systems, providing an opportunity to collectively capture a broad picture of antibiotic networks and the presence or absence of information across these networks. Figure 1 summarises our cross-project analysis of (a) the flow of antibiotics and (b) the distribution of endorsed information on antibiotic use. It reflects the low levels of endorsed information among end users.

Diagram developed through reflective workshops involving researchers from AMIS (Uganda), DRUM (Uganda and Malawi), HATUA (Uganda and Tanzania) and SNAP-AMR (Tanzania).

### Documentary analysis and literature review

Documentary analysis of AMR National Action Plans, policy documents on antimicrobial use, pharmaceutical regulations and historical material on regulation of antibiotics and the medical and veterinary professions across the three countries was carried out alongside the workshops to contextualise findings. The documentary analysis focused on summarising the AMR National Action Plans in each country, with a specific focus on how these address antimicrobial usage, a description of the history of regulation as well as current regulation of antibiotics and the history of the medical and veterinary professionals in the three countries. In addition, we reviewed anthropological literature on antibiotic information, including the enclaving of medical knowledge – that is the gate-keeping within particular realms not generally accessible, of where medical knowledge sits, and how that works transnationally in global health. We reviewed literature on how biomedicine and veterinary medicine has evolved in Uganda, Tanzania and Malawi and examined studies that focused on the provision of information on medicine use to end users.

### Workshops

We conducted workshops with key medical and veterinary professionals, policy makers and drug regulators to understand the factors shaping the flow of antibiotic drug information to end users and identify where interventions could support provision to end users of essential information about antibiotics.

Six three-hour workshops were conducted. In each of the three countries, one workshop discussed antibiotic information in human health and the other animal health. Each workshop was attended by 7 to 10 healthcare professionals, pharmaceutical regulators and policy makers from either human or animal health. These included representatives of the medical and veterinary profession, district health and veterinary officers, drug regulatory bodies, pharmaceutical association, the veterinary association, the AMR Policy Platform and policy makers with an interest in AMR including representatives from the Ministry of Health and Ministry of Agriculture. Ethical approval for the workshops was granted through each of the consortium studies in Uganda, Tanzania and Malawi. Table 1 shows a breakdown of the total number of stakeholders that attended each workshop. A topic guide developed through an iterative process based on reflective workshops was followed, with the research team opening with an explanation of their observation of a gap between spaces of antibiotic information and spaces of antibiotic usage, sharing the mapping graphic shown in Figure 1. The participants were then encouraged to discuss this observation and problem-solve ways to address it. Workshops with human health representatives were given copies of the Coartem (Novartis-produced artemether lumefantrine) packaging that uses pictorial images to encourage end users to complete doses, communicating the need to completely kill all parasites over three days. Workshops with animal health representatives were given copies of an example an information leaflet with pictorial images on the diagnosis and treatment of bovine trypanosomiasis which had been provided to cattle farmers in south Mali [14]. These materials were used to stimulate discussion around models that could be acceptable for sharing antibiotic use information with end users in the three countries and if not where the roadblocks might be and how these could be overcome.

**Table 1.** Stakeholders attending workshops across the three countries.

Category	Uganda	Tanzania	Malawi	*TOTAL*
Human Health	7	7	7	*21*
Animal Health	8	5	10	*23*
Total number of participants	15	12	17	*44*

### Data management and analysis

For the documentary analysis, text from the documents and other materials was summarized systematically under pre-defined dimensions anticipated to shape the sharing of information on antibiotic use, which allowed for direct comparisons across topics and countries [28]. The workshop proceedings were analysed through multiple stages: familiarisation with the data by re-listening to audio recordings and reading and checking transcripts; generation of initial codes to develop a coding frame emergent from the data and comparison between different types of participant and countries to understand differences in contributions. The workshop findings were interpreted in light of the documentary analysis, situating the discussions in the context of historical, national and transnational issues, following established mixed methods analytical processes [29]. The emerging analysis was presented and refined at a series of cross-project multi-disciplinary researcher meetings. Coding and analyses were carried out using qualitative data analysis software, NVivo 12 (QSR International).

### Patient and public involvement

End users and farmers were engaged in the primary studies that were conducted in Uganda, Tanzania and Malawi through participation in medicine surveys, focus group discussions and participant feedback meetings to share antibiotic use experiences. These dialogues with participants informed the questions and approach taken in the workshops described in this article.

## Results

First, we present the findings emerging from the policy documents review of the context regarding access to antibiotics, access to antibiotic use information and regulation of antibiotics across the three countries. Second, we describe the views of our respondents in the workshops regarding the information on antibiotic use that they considered suitable and unsuitable for end users to receive, and the rationales given for this. We share findings on who they considered responsible for giving this information, the suitable channels for sharing information and considerations they discussed in achieving this.

### The human and animal health system and workforce in Uganda, Tanzania and Malawi

In this section, we describe the structure of the human and animal health systems in Uganda, Tanzania and Malawi, followed by a description of key features of human (Table 2) and animal (Table 3) health professionals in the three countries.

**Table 2.** Structure of the human health professions in Uganda, Tanzania and Malawi.

	Uganda	Tanzania	Malawi
Numbers	According to the Ministry of Health [38], there were a total of 66,589 health workers with active licenses in 2020 (1,300 specialist doctors/dentists, 3124 general doctors/dentists, 900 pharmacists 33,420 nurses 14,961 midwives and 12,884 allied health professionals). Overall staffing levels in the public health facilities is at 74%, with the Regional Referral Health facilities having the lowest staffing at 69%.	The number of health care workers increased from 29,063 in 2006/07 to 102,919 in 2019. According to the staffing levels guideline (2014), the number of health workers available is 63,447 and the shortage is 82,007, which is about 56.38%.	In 2016, the Ministry of Health had an estimated 23,188 professional health workers (out of a total of 42,309 positions listed in the MoH staff establishments) working in the public health sector, a 45% vacancy rate. For frontline categories of clinical staff only 17,298 positions were filled of 25,755 positions for both the Christian Health Association of Malawi and the Ministry of Health.
Training	Uganda has a total of 156 registered health training institutions. The institutions offer health related courses, which include a) Medicine and Surgery, Nursing, Public Health, Pharmacy and clinical medicine. Of the 156 health training institutions, 130 belong to the private sector [38].	There are 153 registered training institutions, which offer training programs for health and social welfare workers including:	The Kamuzu University of Health Sciences offers a 5-year program that leads to a Bachelor Medicine and Bachelor of Surgery (MBBS) degree. The College of Medicine runs a 4-year postgraduate program that leads to a Master of Medicine degree in one of the following specialities: Internal Medicine, Paediatrics and Child Health, General Surgery, Anaesthesia and Ophthalmology. The College has since added a degree in Orthopaedics [39].
		(a) Medical Doctor - Five years training + one-year internship.	
		(b) Assistant Medical Officer - Clinical officer + two additional years of rotations/coursework	
		(c) Clinical Officer -Secondary school + three years of medical training.	
		(d) Nurse Midwife -Secondary school + two years of nursing training	
		(e) Clinical Assistant/Medical Assistant ± Secondary school + basic triage skills	
		(f) Village Health Worker/Medical Attendants Short courses on health education, often no formal schooling.	
Structure of professional bodies	The health sector has four professional councils instituted by statutory instruments including:	There are different medical bodies based on profession. These include:	The professional bodies that provide oversight of the private and the public health workers include:
	(a) The Medical and Dental Practitioners Council. Their mandate is to exercise general supervision and disciplinary control over medical and dental practitioners.	(a) Tanzania Nursing and Midwifery Council that regulates and controls Nursing and Midwifery Education and Practice.	(a) Medical Council of Malawi, that regulates doctors, dentists, clinical officers and other paramedical staff excluding nurses. It also issues licenses to all health facilities and regulates training.
	(b) Nurses and Midwives Council that sets and regulates standards of training and practice, registers nurses and midwives and provides professional guidelines for public safety.	(b) The Medical Council of Tanzania, which regulates Medical, Dental and Allied Health practice ensuring high standards, safety and ethical practice through appropriate registration and licensing.	(b) The Nursing Council, that regulates the practice and training of nurses and midwives.
	(c) Allied Health Professional Council whose function is supervising the registration and licensing of Allied Health Professionals and regulating their conduct.	(c) The Pharmacy Council whose mandate is to safeguard and promote the provision of pharmaceutical services to achieve definite therapeutic outcomes for the health and quality of life of clients and promote rational use of medicines.	(c) The Pharmacy, Medicines and Poisons Board, that regulates pharmacy practice and training for pharmacists, pharmaceutical technologists and pharmacy assistants and also regulates pharmacy businesses, including chemists/drug stores, wholesaler/distributors/importers and manufactures.
	(d) The council of the Pharmaceutical Society of Uganda whose mandate is to regulate the practice by pharmacists and their assistants.		(d) The Pharmacy and Medicines Regulatory Authority, that regulates Medicines, allied substances and the exercise of the pharmaceutical profession.

**Table 3.** Structure of animal health professions in Uganda, Tanzania and Malawi.

	Uganda	Tanzania	Malawi
Numbers	There are about 670 registered veterinarians in Uganda and 1,500 paraprofessionals who offer veterinary care for most livestock keepers and owners of companion animals especially in the rural areas [40] There are 27 veterinary staff employed by the government including 12 Animal Husbandry Officers and 15 Veterinarians (personal communication) There are large numbers of informal paraprofessionals.	There are 1021 registered veterinarians and 4118 veterinary paraprofessionals in Tanzania (Veterinary Council of Tanzania, unpublished report). There are large numbers of informal paraprofessionals.	There are 72 Veterinarians at degree level, and 724 Veterinary paraprofessionals (Assistant Veterinary Officers) at Diploma level [23]. There are large numbers of informal paraprofessionals.
Training	Veterinary field officers/field veterinarians (Public and Private) have a degree in veterinary medicine [31]. Since the 1990s, there has been no formal institution providing paraprofessionals with specific training in veterinary medicine in Uganda. Due to their current unregulated training, paraprofessionals’ knowledge varies greatly: some hold a certificate or a diploma in general agriculture or animal management, while others have only had a few months’ training through an NGO or no relevant training at all [31].	Training of all the levels of animal health professionals in Tanzania ranges from Certificate in Animal Health and Production, Diploma in Animal Health and Production to Bachelor of Veterinary Medicine. According to the Tanzania Veterinary Act of 2003 Veterinary paraprofessionals are certificate or diploma holders in animal health, recognized by the Veterinary Council of Tanzania, and practice at all times under the supervision of a veterinarian or veterinary specialist [41]. In practice, many are not trained or registered. Veterinary paraprofessionals formal training curriculum indicates that minimal information is given on antimicrobial use, usually limited to prescribing a few antibiotics, mainly tetracyclines, and there are no organized post-training seminars/workshops [35].	Veterinarians train at Degree level that takes 5 years of training and Veterinary paraprofessionals (Assistant Veterinary Officers) are at Diploma level that takes 3 years of training (personal communication). Informal paraprofessionals may receive training from NGOs or learn on-the-job.
Structure of professional bodies	Uganda Veterinary Association (UVA) is a legally registered professional association with a membership of more than 987 veterinarians. UVA activities are managed by a team of 10 members. UVA engages in community development programmes, animal welfare, policy advocacy, promotion of professional standards and welfare of its members [42] The Uganda Veterinary Board is the profession regulatory body established by an Act of Parliament (The Veterinary Surgeons Act 1958, Cap 277). It is composed of a team of seven veterinarians appointed by the Minister of Agriculture, Animal Industry and Fisheries with approval of Cabinet. Its major mandate is to ensure that animal health services are offered by qualified, registered and licensed veterinary professionals under their regulatory supervision. (www.ugandavetboard.org)	Tanzanian Veterinary Association originated from the Tanganyika Territory Division of the National Veterinary Medical Association (NVMA) of Great Britain and Ireland. Later on, the National Veterinary Medical Association gave birth to the British Veterinary Association (BVA) and the Association in the then Tanganyika Territory became the Tanganyika Division of the British Veterinary Association. In the 1950s, members of the Tanganyika Division of the British Veterinary Association had to be full members of the British Veterinary Association in the first place. Since the Union of Tanganyika and Zanzibar in1964, the association is known as Tanzania Veterinary Association (TVA) [43].	The Malawi Veterinary Association is an active body and all veterinarians are encouraged to join. It promotes professionalism, ethics and protects the interests of the profession. It has a three-member executive body. The profession is regulated by the Veterinary and Para-Veterinary Practitioners Act which is yet to be operationalized (Personal communication).

#### Uganda

The human health system in Uganda is a mix of public and private health care providers. The public health system in Uganda is multi-layered including Health Centre IIs at parish level, Health Centre IIIs at sub-county level, Health Centre IVs at county level, and higher-level district hospitals, regional referral hospitals and a national referral hospital. The private sector consists of pharmacies, drug shops, private clinics and private hospitals and the private not for profit health care providers [30]. Veterinary services are organized under the central government and the local government. Veterinarians and paraprofessionals employed by the government provide preventive services free of charge. However, when they provide clinical services, these are paid for by farmers. The veterinary sector is predominantly private with animal health services provided by veterinarians and paraprofessionals [31].

#### Tanzania

The human public health care system in Tanzania is hierarchical, consisting of dispensaries, health centres, district hospitals, regional hospitals and tertiary hospitals at zonal and national levels [32]. Private health care providers in Tanzania include hospitals, health centres, dispensaries, clinics, laboratories, pharmacies, Accredited Drug Dispensing Outlets and maternity homes. Veterinary services in Tanzania are within the Ministry of Livestock and Fisheries Development and have a central component under the Director of Veterinary Services and a regional component under the local authorities [33]. Animal health services in Tanzania are offered by livestock field officers employed by the Tanzanian Ministry of Livestock and Fisheries, paraprofessionals and agrovet shops where veterinary medicines are sold [34]. Most livestock keepers buy drugs to treat their animals from drug shops run by informal paraprofessionals [35].

#### Malawi

Malawi’s health system is organized at community, primary, secondary and tertiary level linked to each other through an established referral system [36]. At community level, health services are provided by health surveillance assistants, health posts, dispensaries, and maternity clinics. At primary level, health services are provided by health centres and community hospitals. The secondary level of care consists of district hospitals and Christian Health Association of Malawi hospitals of equivalent capacity. The tertiary level consists of central hospitals that provide specialist health services at regional level. Health services in the public sector are free-of-charge while user fees have to be paid by patients for Christian Health services [37]. The private for-profit sector consists of private hospitals, clinics, laboratories and pharmacies. Traditional healers are also prominent. The private not-for-profit sector comprises religious institutions (some of which are integrated into the public provision), non-governmental organisations, statutory corporations and companies. Veterinary Services in Malawi are under the Ministry of Agriculture and are centralized, with no devolved functions [33]. Informal paraprofessionals are important providers of veterinary services.

### Unrestricted access to antibiotics amidst scarcity of information on antibiotic use

Expert participants in the workshops across the three countries agreed that antibiotics were widely accessible and yet information on antibiotic use was scarce. Beyond public and private health facilities, end users were recognised to obtain antibiotics from pharmacies and drug shops with and without prescription. Participants across the three countries described the primary sources of information currently provided to the public on use of medicines as being through medicine package inserts, health care providers and media campaigns. However, package inserts were described as excluding many end users who were unable to read and/or understand English or medical jargon. Participants reported that the information on antibiotics that health care providers shared with patients during their encounters was to emphasise the need for patients to complete the doses of medicines prescribed and communicate that some diseases do not require antibiotic treatment. In Uganda, the National Drug Authority (NDA) was using local media to pass on messages to the general public discouraging undesirable practices such as self-prescription of antibiotics while encouraging good practices such as taking antibiotics as instructed by healthcare providers. In Tanzania, the pharmacy council had developed leaflets for the general public on irrational use of medicines and the likely consequences. The quotes below are indicative of the view held by the majority of those at the workshops with regard to the kind of information on antibiotic use that was being shared with the general public.

Now I can go to the technical people, and I give them as much information but when I go to the public there is a limit to what I give and yes, we talk about these antibiotics many a times we don’t talk about specific antibiotics. But I talk about when I give you this medicine, if they tell you ‘take it like this please try and make sure you take it like that because if you don’t do that, this is what may come out of that.’ I will tell them, ‘if you use this antibiotic without getting it prescribed by an authorised person, this is what might … … this is what is going to happen to you.’ So, I will talk about the dangers. I will talk about how to use, how to store and how to dispose. (Human Health professionals Workshop, Uganda)

Yeah, so you have to just provide information that to a layman person it’s going to make sense and also, they need to understand that they are taking an antibiotic, let us say they have a bacterial infection, and they are being given azithromycin, you have to be told, let’s say you have this infection, and we are giving you an antibiotic, ‘what you are taking is an antibiotic. If you don’t follow the dosage, there is resistance.’ (Human Health professionals Workshop, Malawi)

In the veterinary sector, some participants reported that information on antibiotic use for animal treatment was available in medicine packs but was not well understood by most farmers, who they said were typically illiterate. In Tanzania, the Food and Agricultural Organisation had developed treatment guidelines including information for farmers on the management of diseases in poultry. In Uganda, the NDA was sharing information with farmers through electronic and print media with a focus on addressing AMR and encouraging farmers always to seek the services of trained veterinary professionals. Unrestricted access to antibiotics for animal treatment was reported across the three countries. This was happening amidst complex dynamics around veterinarians. In Uganda, veterinarians were described as not empowered enough to implement laws on antibiotic use because of gaps in the existing policies and laws. Participants reported that with many unqualified professionals offering veterinary services in Tanzania, the trained professionals could hardly compete with them in the farming communities.

### Gaps in implementation of antibiotic regulation across Uganda, Tanzania and Malawi

Enforcement of antibiotic regulations was described as lacking across the three countries. In Malawi, participants expressed concerns that licenses were being issued to unqualified people. Malawian participants suggested that organisations responsible for handling the regulation of pharmaceuticals were poorly coordinated; for example, the Ministry of Trade issued licenses without consulting the department of Animal Health and Livestock Development or the Poisons and Medicines Regulatory Authority. Further, despite the law stating that clinics should not run dispensaries if they are within a radius of 5 km from a pharmacy, this practice continued. At the time when we held the workshop in Malawi, the quality of veterinary medicines was not being assessed or approved and veterinary medicines were not being registered. Workshop participants expressed concern that with gaps in enforcement of regulations and porous borders, people were at liberty to import any veterinary medicines they wanted without a permit. In Uganda, respondents reported efforts were being made to pursue the enforcement of regulation and that the NDA were working with local authorities to ensure enforcement of regulation and to support supervision and compliance monitoring. It was also reported that in Uganda, the NDA was also withdrawing their license and medicines from pharmacies and drug shops they found run by unqualified people. The NDA officials also reported engaging in efforts to withdraw antibiotics from the public eye as a measure to minimise their demand by the general public. Similarly, in Tanzania participants reported that the Accredited Drug Dispensing Outlets were poorly supervised by the local government and the pharmacy council that over saw them.

### Patients and farmers should receive only limited antibiotic information only

At one of the workshops, a veterinary professional expressed his fears around farmers being equipped with detailed information on use of antibiotics:

There is a very big danger with having, arming half-baked people with weapons. Drugs are weapons that can kill and if they are in the hands of half-baked people you are making them be in danger. (Animal Health Professionals workshop, Uganda)

Whilst this respondent’s language was pointed, his core concerns were voiced repeatedly by workshop participants across all three countries. There was a widespread concern that detailed information on use of antibiotics, including the types, doses and the conditions that can be treated with antibiotics, should not be shared with end users. Experts at the workshops consistently felt that both patients and farmers tended to desire more information than they required. They felt it was not the end user’s role to diagnose conditions that they or their animals suffer from. Respondents felt strongly that the end users’ role was to present to the places where qualified expert health providers could make those assessments and provide prescriptions, with information on, what by implication is, a need-to-know basis.

Participants justified this strongly-held belief on the basis that end users were incapable of properly assimilating information on the use of medicines. Some of the reasons that were given against sharing detailed information with end users included that there are many types of antibiotics, with different dosages and uses for different ailments, all of which had the potential to confuse end users. Farmers were described as being incapable of making a differential diagnosis between conditions with similar symptoms or of fulfilling other conditions for determining appropriate treatment. Participants were of the clear opinion that sharing detailed information on antibiotic use could lead farmers and patients to believe that they themselves were health experts. This proposition was often met with incredulity by the expert participants,

Now, are we going to say that our farmers should treat; are farmers to be doctors themselves? Are we going to say so? Are we going to say that? (Moderator: That they are doctors?)

Yes, they should come to be doctors themselves for their animals because if I am saying use this dose, you see, of injectable antibiotics so, that means that you don’t go to a doctor but use this. (Veterinary professional - Animal Health Professionals Workshop, Tanzania)

Experts’ concerns appeared to be two-fold. First, professionals were concerned that sharing detailed information on antibiotic use would likely increase self-medication and disease mismanagement in both humans and animals, both of which could further drive AMR. Second, experts feared the impacts on their professions. Notwithstanding the many vacancies in the public health sector and the extreme dearth of veterinary professionals, respondents were keen to point out that large numbers of trained human and animal health workers graduated from academic institutions every year across the region. Giving farmers and patients detailed information on antibiotic use threatened to undermine the role of trained animal health workers. Interestingly, in Malawi some participants felt that farmers could be trusted with administering medicines that came in powder form but not those that came in injectable form. Other participants in the same workshop contested the competency of farmers to administer powdered antibiotics, suggesting they would likely use the wrong ratios.

### End users and farmers must only be given general guidance on use of medicines

Although there was a general reluctance to share information, some participants acknowledged that it was necessary to share information on antibiotic use with end users because the gaps in enforcement, such as those regulating dispensing, mean that farmers and patients regularly access antibiotics without a prescription and therefore without the (often verbal) instructions that health experts suggest they normally give when prescribing. This said, experts across the three countries only slightly modified their primary view by saying that any such information provided to end users should be of a very general nature and focused on how to use medicines;

I think it’s better we go with the general [approach] that tells people how to respect drugs in general because we’ve already hinted at this point, they [antibiotics] are as varied as [anything] … . and even for me the medic, sometimes I have to open and look at the [instructions for a] new antibiotic, so for the community they are just going to be confused. They just have to learn that with medicines, these are the general principles. (Human Health Professionals Workshop, Uganda)

Further discussion during the workshops led to idea-generating sessions on what might be included if more general information about medicines were delivered to end users. Results are listed in Table 4. Notably, most of the information that respondents proposed should be shared with end users fell in the category of instructions on appropriate behaviour rather than in the category of explaining why it would be important to adopt certain practices and the likely benefits.

**Table 4.** General information about medicines that expert respondents believed should be shared with end users.

Sector	Type of information deemed suitable for sharing with end users
Human treatment	Directive Instructions:
	● To avoid sharing medicines
	● To avoid stocking medicines
	● To avoid taking medicines left over from previous illness episodes
	● To always seek services of trained health professionals
	● To follow health worker instructions on the dose and duration of treatment
	Specific advice about the given drug
	● Possible side effects
	● Drug interactions
	● Dietary requirements when on medication
	● How to store medicines
	● How to dispose of medicines not taken with in the recommended period
	General Advice about health and drugs:
	● Where to access good quality medicines
	● Who is allowed to sell medicines
	● What to expect from places where healthcare is accessed
	● Questions to ask the person selling you medicines
	● To return to the healthcare provider if there is no improvement
	General warnings
	● Consequences of misusing medicines
	● The danger of antimicrobial resistance
Animal treatment	General information to help determine if an animal is sick and where to seek help
	● Signs and symptoms of diseases
	● Sources of genuine veterinary services
	Directive instructions
	● To consult veterinary professionals when their animals present certain signs and symptoms
	● To only give antibiotics under the supervision of a veterinary professional
	● To avoid using antibiotics for growth promotion
	● To avoid using antibiotics for prevention of diseases
	Information on reducing the need for antibiotics
	● Disease prevention methods
	● Hygiene and biosecurity to minimise the need for antibiotics
	● Encouraging the vaccination of animals
	● Promoting the use of alternatives to antibiotics
	● Promoting good practices at all steps of production and processing of food from animals
	General warnings
	● Consequences of the misuse of medicines (AMR)
	● Antibiotics do not treat everything

### Detailed antibiotic use information should be shared only with trained human health and veterinary professionals

Aside from the general information that might be given to end users, study participants across the three countries remained committed to the idea that access to detailed antibiotic use information should be kept within the confines of trained professionals, as explained by this veterinary professional,

Having said that, the context in our country what is closest to doing such a thing [sharing detailed antibiotic use information] will not reach the farmer, at worst it is going to work with the community-based animal health workers who have a specific area of jurisdiction and who are assigned specific responsibilities under a trained professional. (Animal Health Professionals workshop, Uganda)

Beyond trained professionals, participants were of the view that this information might be entrusted with community health workers only if they would undergo some training and receive close supervision. Participants suggested that existing community health structures could be used to share the information – including health educators, community health workers, village health committees or teams, Health Surveillance Assistants, Community Health nurses, animal extension workers and assistant veterinary officers. The community health care system as a channel for disseminating messages was described as follows;

Like he said we have a well-structured community health care system where when the information reaches the district health facility that information is dispensed further to the health centres and the assumption is that each and every community at least has a health centre and if they don’t have that I also know that each community has a local village health committee, and these local village health committees work with health surveillance assistants and these report to the DHO. (Human Health Professionals Workshop, Malawi)

In a hospital setting, the prescriber and dispenser were seen as key people responsible for passing on information on the use of medicines to end users. Participants pointed out that messages on antibiotic use could be incorporated into information sharing structures at the health facilities such as during antenatal clinics and family planning visits. Farmer associations and clubs were also suggested as a channel for sharing information on antibiotic use with farmers. Leaders such as religious and political leaders and schools were also suggested as channels for sharing (limited) information on antibiotic use with end users.

## Discussion

With increasing concerns about AMR, the focus of communication campaigns over previous decades has been to raise awareness among end users about the dangers of misusing antibiotics, and more recently the dangers of AMR. Despite this, the consumption of antibiotics purchased over-the-counter in LMICs continues to be high [10,44] including for animal use [23,45]. Our previous research observed a gap at the point of antibiotic use of information on recommended use as agreed by medical and veterinary bodies, and a desire by end users to access this detailed information [20–27]. In this study, findings from our workshops with policy makers, drug regulators and health care professionals revealed that the appetite of these regulatory and professional bodies for devolving antibiotic use information to end users was low. This was rationalised by concerns that information on medicine use is too complicated to be understood by lay people, underpinning a rhetoric that information be ‘safeguarded’ within the confines of trained professionals. This meant that information to be shared with end users was considered best formatted in instructive messages, particularly to follow professional advice. We argue that not only is such enclaving of knowledge a common feature of technocratic and professionalised Western medicine but in this context, is also rooted in the post-colonial histories of Uganda, Tanzania and Malawi. By making visible the social position of antibiotic information among health care professionals, this paper demonstrates tensions that must be addressed while a large number of patients and farmers remain without good access to clinical and veterinary professional services. We propose a move towards point-of-care information about antibiotics from unbiased sources through a client care-centred approach.

### Locating antibiotic information

Understanding the physical and social location of antibiotic information, and the potential for this to change, revealed professionals and regulators to be positioned between responsibilities to protect the public and responsibilities towards universal health coverage. One way this was navigated was to draw upon established models of lay ignorance; end users would not be in position to fully understand medical information, carry out differential diagnosis and appropriately use medicines, putting themselves and others at risk. Under the auspices of altruistic paternalism, the perpetuation of a lay ignorance model [26,46] promotes the gatekeeping role of medical professionals. The idea that biomedical knowledge should be kept within the confines of trained professionals has been well described as part of the esoteric knowledge which meant that professionals were experts in relation to their clients, and able to maintain control and autonomy [47]. This positioning was compounded with colonial medicine, as observed in East Africa where medical training was undertaken by medical missions and could only be accessed by a special category of the population [48]. Even with their extensive stay in Africa, few of the colonial authorities who held much of the medical knowledge spoke local languages and instead identified local elites familiar with western medicine to act as intermediaries in dispatching certain medical knowledge but not medical practice [49]. While the practice of restricting medical knowledge in part emerged from genuine concerns for safety, it was also a way to enclave medical knowledge and retain power.

Technical language has been observed to be used as a tool for keeping medical knowledge exclusive to medical professionals [50,51] and the use of Latin in medical texts and case reports may on the one hand serve the purpose of standardising communication among medical professionals worldwide [52] but also reinforces the elite enclaving of medical knowledge. This situation is not unique to the Western biomedical tradition; the mystification of healing knowledge has been observed across a variety of healing traditions around the globe [53,54]. Despite a shift in recent decades towards patient-centred care, this study suggests it will continue to be a challenge for health care professionals to agree to non-medically trained people being given detailed information on medicines in these established social domains [55].

However, there is evidence that drug sellers, livestock keepers and informal paraprofessionals can succeed at identifying conditions for which they can administer treatments themselves [56,57]. Pilot interventions conducted in Mali, England, Kenya and Tanzania have reported potentially positive impacts of providing medicine use information to end users, suggesting that provision of targeted information to patients and farmers through simple strategies such as leaflets can result in safe use of medicines [14–17].

### Communicating antibiotic information

Our study participants preferred messages to the public that emphasised compliance with professional instructions coupled with information about the negative consequences of non-compliance, including antimicrobial resistance. We observe that a similar compliance-oriented approach is reflected in the awareness strategies of global health actors to counteract AMR [58], which have mobilised apocalyptic narratives that conceptualise antimicrobial ‘misuse’ – among both humans and animals – as a global ‘threat’ leading towards what many have termed a ‘post-antibiotic era’ [59,60]. Awareness campaigns with the general public have demonstrated limited success, despite being core to the strategies being deployed to address AMR [6,61–63].

This mode of messaging can be understood as a ‘doctor-centred’ rather than ‘patient-centred’ model of care [64]. If the needs of patients, carers and farmers seeking care were centred, not only would the use case for antibiotic information be reorientated towards actionable problem-solving, but the needs for the wider care system for both people and animals would be foregrounded. Attempts to share information with end users in biomedical practice have also been achieved through patient-centred care practices [65], although with some limitations emerging from language differences, differences in vocabulary, age, background and familiarity with medical technology [66].

We propose point-of-care information about antibiotics as a client care-centred approach. In line with the WHO ambition to establish ‘effective medicines information systems to provide independent and unbiased medicine information … to the general public’ [67], the source of such information cannot only be the commercially driven pharmaceutical advertising strategies on which many professionals and clients currently rely on. The WHO Essential Medicines List Antibiotic Book, launched in 2022 and aimed at improving antibiotic awareness, is one such source [68] and in in our particular study countries, the African Medicines Authority, ratified in 2021, also presents an opportunity for a coordinated regulation and information approach.

### Limitations and future research

Our research focused on three countries in which the authors have carried out long-standing research into antimicrobial medicines across the health systems. While the workshops reported here round-out our analyses by providing high-level perspectives of those with authority in the human and animal health sectors, it is possible that additional research – for example with donors of medicines and with private sector actors – could have expanded our understanding of the phenomenon of antibiotic information flows. Following from the findings and analysis in this paper, future research could focus on the structures that are maintained through the enclaving of knowledge and consider the consequences of potential disruption, alongside designing and evaluating pilots to shift towards a client-centred model of unbiased antibiotic information at the point-of-care.

## Paper context

AMR action plans prioritise raising public awareness of AMR, including to reduce antibiotic use. Our previous research in Uganda, Malawi and Tanzania showed demand amongst patients and farmers for information about using antibiotics. This study’s workshops with members of regulatory and professional bodies in these countries demonstrated low interest in devolving antibiotic information to end-users, under the rhetoric of safeguarding. Without good access to prescribers, the tension between enclaving and sharing of knowledge presents an equity issue.
